# Neonatal Immunology

**DOI:** 10.3390/ijms25179395

**Published:** 2024-08-29

**Authors:** Michael Zemlin, Christoph Härtel

**Affiliations:** 1 Department for General Pediatrics and Neonatology, Saarland University Medical Center, 66421 Homburg, Germany; 2 Center for Digital Neurotechnologies Saar (CDNS), 66421 Homburg, Germany; 3 Center for Genderspecific Biology and Medicine (CGBM), 66421 Homburg, Germany; 4 Department of Pediatrics, University of Wuerzburg, 97080 Würzburg, Germany; haertel_c@ukw.de; 5 International Research Training Group 1911, University of Lübeck, 23538 Lübeck, Germany; 6 German Center of Infection Research, Hamburg-Lübeck-Borstel-Riems, 23538 Lübeck, Germany; 7 Interdisciplinary Center of Clinical Research, University of Würzburg, 97080 Würzburg, Germany

*“There can be no keener revelation of a society’s soul than the way in which it treats its children.” —Nelson Mandela*

Immunologically, the neonatal period is unique in several aspects, including but not limited to first exposure to environmental antigens such as microbes and nutrition, the establishment of microbiota, the initiation of secondary lymphatic structures, the presence of maternal passive immunity, and the initiation of immune defense mechanisms. The high plasticity of the neonatal immune system goes along with a marked vulnerability to lifelong imprinting effects from environmental exposures. A holistic understanding of these delicate trajectories, the infant’s physiological needs, and their impact on long-term health is the key to treating children appropriately. Prioritizing research in neonatal immunity is a great step in supporting the development of children. In this Special Issue, researchers have contributed new insights into neonatal adaptive and innate immunity, mechanisms of neonatal inflammation, microbiota, and infection.

Three publications predominantly address aspects of the neonatal adaptive immunity [1,2,3], four publications address the neonatal innate immunity [4,5,6,7] and one manuscript addresses the interaction between players of the innate and adaptive immunity [8].

Sex differences were often regarded as a potential bias for clinical studies in neonatology, but the reasons for these striking differences remain largely unknown. It is well established that premature and term-born girls have a significantly lower mortality and long-term morbidity than boys. Bous et al. present analyses of multiple B-cell and T-cell subpopulations in human cord blood showing that B1-cells, marginal zone memory T-cells, and naïve thymus negative Th-cells are less abundant in female than in male cord blood [1]. Characterization of sex differences in various physiological systems might prove to be a major step towards more individualized preventive and therapeutic strategies.

Worldwide, sepsis is a major killer of babies and a significant contributor to long-term morbidity. Consequently, combating neonatal sepsis is a sustainable developmental goal of the WHO and is regarded as a primary research aim for neonatal care societies. Despite great progress in clinical treatment, the molecular mechanisms of neonatal inflammation remain poorly understood, and thus, the medical interventions primarily remain confined to symptomatic treatment. Apparently, it is more the immunological framework of the neonate than the individual actors of the immune system that cause this so called “immunodeficiency of immaturity”. Interestingly, Majer and colleagues found that despite the absence of memory T-cells, the unexperienced neonatal CD4 T-cells are able to mount robust anti-bacterial responses that are regulated by the PD/PD-L1 axis [2]. Two publications address the molecular mechanisms in necrotizing enterocolitis: The role of regulatory T-cells in necrotizing enterocolitis (NEC) was reviewed by Zuiderwijk et al. [3]. The authors conclude that further studies are needed to clarify whether the decline in regulatory T-cells and the increase in Th17-cells during NEC development reflects a causal relationship or an epiphenomenon [3]. Schoenaker et al. performed a proof of principle showing that a novel technology, epigenetic immune cell counting, can be used to quantify lymphocyte subpopulations from dried blood spots in newborn screening cards [6]. They found significant differences between preterm and term cord blood, but not between age-matched preterm groups with or without later development of NEC. Interestingly, the number of regulatory T-cells was similar in all three study groups.

The rapid expansion of the microbiota and its mutual interaction with the immune system are hallmarks of the first postnatal days and weeks. The host’s immune system serves as a “gardener” for the developing microbiota, maintaining a balanced ecosystem, while the microbiota educates the immune system. Marißen et al. provide an in-depth review of current knowledge on microbiota-regulating peptides/proteins (MRPs), also known as antimicrobial polypeptides, in neonates [7]. Research on MRPs is urgently needed as they could be useful in preventing and treating infections. This publication highlights the importance of addressing gestational age and postnatal age in future strategies aimed at modulating the immune system for the benefit of individual children.

In a mouse model, Rühle et al. studied the collaboration between innate and adaptive immune systems: They found that newborn mice being depleted from Ly6G-expressing neutrophils exhibit an altered peripheral T-cell homeostasis with a decreased CD4+/ CD8+ T-cell ratio and increased numbers of CD4+/CD8+ double positive thymocytes [8]. The authors conclude that they identified a previously unknown mechanism mediating the immunomodulatory effects of neutrophils in newborn mice. In a concise review, Renske de Jong et al. present insights in immunometabolism in neonatal monocytes and macrophages [4]: Multiple pathways are involved in the altering metabolic status of monocytes and macrophages after stimulation by (extrauterine) antigens, e.g., glycolysis, the tricarboxylic acid cycle and oxidative phosphorylation. These insights help to understand the mechanisms of the so called “disease tolerogenic state” of the fetus and the switch towards immune defense after exposure to the extrauterine environment.

Inflammatory cascades are crucial not only in infections, but also in other conditions such as mechanical trauma (e.g., mechanical ventilation) or cerebral bleeding. Alshareef et al. demonstrate in a mouse model that the inflammation associated with germinal matrix hemorrhage is driven by complement [5]. This confirms clinical experience that the occlusion of the cerebral aqueduct and the excessive production of cerebrospinal fluid is driven by inflammation.

In conclusion, the results reported in this Special Issue on neonatal adaptive and innate immunity and their crosstalk shed light into basic mechanisms which are unique to the neonatal adaptive and innate immunity.

This knowledge may further contribute to understanding the mechanisms of immunological imprinting of chronic inflammatory diseases such as allergies and autoimmunity and how to use the perinatal “window of opportunity” to reduce the risk of chronic disease. Since the diversification of the neonatal immune system is paralleled by the fulminant expansion of the microbiota, the latter might be a key to prevention and therapy. Understanding neonatal immunity can reveal novel approaches in modulating developmental trajectories to the benefit of everyone.

## References

[1] Bous M., Schmitt C., Hans M.C., Weber R., Nourkami-Tutdibi N., Tenbruck S., Haj Hamoud B., Wagenpfeil G., Kaiser E., Solomayer E.F. (2023). Sex Differences in the Frequencies of B and T Cell Subpopulations of Human Cord Blood. Int. J. Mol. Sci..

[2] Majer C., Lingel H., Arra A., Heuft H.G., Bretschneider D., Balk S., Vogel K., Brunner-Weinzierl M.C. (2023). PD-1/PD-L1 Control of Antigen-Specifically Activated CD4 T-Cells of Neonates. Int. J. Mol. Sci..

[3] Zuiderwijk M.O., van der Burg M., Bekker V., Schoenaker M.H.D. (2022). Regulatory T Cells in Development and Prediction of Necrotizing Enterocolitis in Preterm Neonates: A Scoping Review. Int. J. Mol. Sci..

[4] de Jong R., Tenbrock K., Ohl K. (2023). New Insights in Immunometabolism in Neonatal Monocytes and Macrophages in Health and Disease. Int. J. Mol. Sci..

[5] Alshareef M., Hatchell D., Vasas T., Mallah K., Shingala A., Cutrone J., Alawieh A., Guo C., Tomlinson S., Eskandari R. (2023). Complement Drives Chronic Inflammation and Progressive Hydrocephalus in Murine Neonatal Germinal Matrix Hemorrhage. Int. J. Mol. Sci..

[6] Schoenaker M.H.D., Zuiderwijk M.O., Bekker V., Bredius R.G.M., Werner J., Schulze J.J., van der Burg M., Blom M. (2023). Epigenetic Immune Cell Counting to Analyze Potential Biomarkers in Preterm Infants: A Proof of Principle in Necrotizing Enterocolitis. Int. J. Mol. Sci..

[7] Marissen J., Reichert L., Haeartel C., Fortmann M.I., Faust K., Msanga D., Harder J., Zemlin M., Gomes de Agüero M., Masjosthusmann K. (2024). Antimicrobial peptides (AMPs) and the microbiome in preterm infants: Consequences and opportunities for future therapeutics?. Int. J. Mol. Sci..

[8] Rühle J., Ginzel M., Dietz S., Schwarz J., Lajqi T., Beer-Hammer S., Poets C.F., Gille C., Köstlin-Gille N. (2023). Depletion of Ly6G-Expressing Neutrophilic Cells Leads to Altered Peripheral T-Cell Homeostasis and Thymic Development in Neonatal Mice. Int. J. Mol. Sci..

